# Variable size computer-aided detection prompts and mammography film reader decisions

**DOI:** 10.1186/bcr2137

**Published:** 2008-08-25

**Authors:** Fiona J Gilbert, Susan M Astley, Caroline RM Boggis, Magnus A McGee, Pamela M Griffiths, Stephen W Duffy, Olorunsola F Agbaje, Maureen GC Gillan, Mary Wilson, Anil K Jain, Nicola Barr, Ursula M Beetles, Miriam A Griffiths, Jill Johnson, Rita M Roberts, Heather E Deans, Karen A Duncan, Geeta Iyengar

**Affiliations:** 1 Division of Applied Medicine, School of Medicine & Dentistry, University of Aberdeen, Lilian Sutton Building, Foresterhill, Aberdeen AB25 2ZD, UK; 2 Division of Imaging Science & Biomedical Engineering, University of Manchester, Stopford Building, Oxford Road, Manchester M23 9LT, UK; 3 Breast Screening Unit, Nightingale Centre & Genesis Prevention Centre, Wythenshawe Hospital, Southmoor Road, Manchester M23 9LT, UK; 4 Department of Public Health & General Practice, Christchurch School of Medicine & Health Sciences, University of Otago, Riccarton Avenue, Christchurch 8140, New Zealand; 5 North West Genetics Knowledge Park (Nowgen), The Nowgen Centre, 29 Grafton Street Manchester M13 9WU, UK; 6 Department of Epidemiology, Mathematics & Statistics, Wolfson Institute of Preventive Medicine, Charterhouse Square, London EC1M 6BQ, UK; 7 North East Scotland Breast Screening Centre, Foresterhill Road, Aberdeen AB25 2XF, UK

## Abstract

**Introduction:**

The purpose of the present study was to investigate the effect of computer-aided detection (CAD) prompts on reader behaviour in a large sample of breast screening mammograms by analysing the relationship of the presence and size of prompts to the recall decision.

**Methods:**

Local research ethics committee approval was obtained; informed consent was not required. Mammograms were obtained from women attending routine mammography at two breast screening centres in 1996. Films, previously double read, were re-read by a different reader using CAD. The study material included 315 cancer cases comprising all screen-detected cancer cases, all subsequent interval cancers and 861 normal cases randomly selected from 10,267 cases. Ground truth data were used to assess the efficacy of CAD prompting. Associations between prompt attributes and tumour features or reader recall decisions were assessed by chi-squared tests.

**Results:**

There was a highly significant relationship between prompting and a decision to recall for cancer cases and for a random sample of normal cases (*P *< 0.001). Sixty-four per cent of all cases contained at least one CAD prompt. In cancer cases, larger prompts were more likely to be recalled (*P *= 0.02) for masses but there was no such association for calcifications (*P *= 0.9). In a random sample of 861 normal cases, larger prompts were more likely to be recalled (*P *= 0.02) for both mass and calcification prompts. Significant associations were observed with prompting and breast density (*p *= 0.009) for cancer cases but not for normal cases (*P *= 0.05).

**Conclusions:**

For both normal cases and cancer cases, prompted mammograms were more likely to be recalled and the prompt size was also associated with a recall decision.

## Introduction

In a breast screening programme, film readers are required to read large volumes of mammograms to detect a relatively small number of cancers (<1.0%). The radiographic complexity of breast tissue, the subtle nature of the mammographic features in early breast cancers and reader fatigue or distraction make this a challenging task [1]. The performance level of readers is known to vary widely between general radiologists and breast imaging experts [2], and even among experienced mammography film readers [3-5]. Retrospective evaluation of interval cancer cases suggest that 16% to 27% of cases show evidence of an abnormality on the prior screening films [6-8], with at least 40% of cases wrongly dismissed as benign or showing no abnormality [9,10]. Some cancers are therefore missed due to visual search errors; other cases are missed as although abnormalities are noted they dismissed by the radiologist [11,12].

Computer-aided detection (CAD) systems have been developed to assist film readers to improve their performance [8,13,14]. CAD uses software-based detection algorithms to identify regions of the mammogram with suspicious features, which will be marked with a prompt. The aim of CAD is to draw the reader's attention to these areas so he/she can decide whether they appear genuinely abnormal. In addition, there is some evidence that CAD could increase the detection of early-stage breast cancers [13,15-18].

The potential benefit of the first generation of CAD systems was somewhat overshadowed by their relatively low specificity, requiring film readers to discriminate between numerous false-positive CAD marks (prompts) and true positives that warranted further evaluation [19-24]. A system with a high false-marker rate is likely to degrade reader performance and would be unacceptable in a screening programme [25-27].

In a small observational study of 120 cases including 44 cancer cases, Taylor and colleagues assessed the impact of CAD on film reader sensitivity and specificity using one of the earlier versions of the R2 ImageChecker, and reported that readers were more likely to respond to emphasised prompts (that is, prompts that the CAD system circled to indicate a higher probability of malignancy) [28]. Recent versions of CAD software now use the algorithm output to produce a measure of the likelihood that a prompt is marking a genuine abnormality. This generates prompts of different sizes, with the largest marking regions where there is the most evidence of abnormality [29-31]. Giving the reader additional information on the likelihood of a prompt marking a malignancy might aid decision-making once a lesion is found.

As part of a retrospective study comparing the cancer detection rate and recall rate of single reading with CAD and of double reading in a large sample of screening mammograms (>10,000) containing relatively few cancer cases [32], we have investigated the impact of prompting and prompt size on reader behaviour.

## Materials and methods

Two ImageChecker M1000 version 5.0 CAD Systems were loaned by R2 Technology Inc. (A Hologic Company, Santa Clara, CA, USA) over a 2-year period. No other financial support was given, and the authors had full control of the study data and information submitted for publication.

Local research ethics committee approval was obtained, and informed consent was not required. All mammograms were anonymised.

### Study design

Two screening centres participated in the study. Mammograms were sampled from a routine screening mammography during 1996 in women aged 50 years or older that had been double read. Mammograms were randomly allocated to be read by a radiologist using CAD who had not been recorded as the first or second reader in 1996 [32].

### Case selection

The dataset consisted of 10,096 mammograms from the 1996 screening round. Study cancer cases (Table 1) were defined as those diagnosed at the original screen, those diagnosed in the 3-year interval following the 1996 screen (interval cancers) or those diagnosed at the next scheduled screen in 1999 (next-screen cancers). Cancers diagnosed after the 1999 screen were also included. Only the mammograms from the 1996 screen were digitised and analysed by CAD. All cancer cases had histological evidence of malignancy present on biopsy or, exceptionally, on cytology, and were verified by local cancer registries. A random sample of 10% of the 9,781 normal cases was used to examine reader behaviour to false prompts and to no prompts.

**Table 1:** Study cases

Case	*n *(%)
Random sample of cases from the 1996 screening round with full screening history available	10,096
Cancer cases	315
Detection status	
Detected at original 1996 screen	85 (27)
Detected as interval cancer (within 1 year)	7 (2)
Detected as interval cancer (2 to 3 years later)	42 (13)
Screen detected at next screen in 1999	96 (31)
Cancer thereafter	85 (27)

### Film digitisation and computer-aided detection analysis

Mammograms were anonymised, digitised and analysed by the CAD system signal-processing algorithms. Prompts were displayed on a flat-panel display screen superimposed on a reduced resolution version of the corresponding mammogram. Prompts were generated by the CAD system algorithms for masses (indicated by asterisks) and for microcalcifications (indicated by solid triangles), and were placed over the lesion in question. On regions where both a mass and microcalcifications were detected, a composite malc marker (indicated by four-pointed stars) was displayed.

The size of the CAD prompts was related to the likelihood of malignancy as determined by the algorithms. In the present study, algorithm thresholds were adjusted to give a detection sensitivity of approximately 85% for masses and 98% for calcifications, with corresponding false-marker rates of 1.5 and 1.0, respectively, per four-film case. The PeerView facility provided readers with a high-resolution image of any regions of interest (ROIs) identified by the CAD algorithm [30].

### Film readers

Four readers from each centre participated in the study. All readers met the quality assurance standards of the National Health Service Breast Screening Program [33], reading an average >5,000 mammograms per year, with 2 to 15 years of screening experience, and having undergone a 2-month training period in the use of CAD prior to the start of the study (consisting of an initial training session from the CAD system manufacturer, followed by consolidation and practice sessions using six training sets of 75 to 100 cases each). The initial sets included 25% cancer cases, and this percentage was progressively reduced to 5% to train the readers to dismiss prompts in a low cancer rate environment as would be encountered in a screening setting [32,34].

### Film reading procedure

In the main study, screening mammograms were randomly allocated to a reader who had not been involved in the original double reading in 1996. The reader first viewed the mammograms and noted any abnormalities *pro forma*. Prior mammograms were viewed if they had been used at the time of the original double reading. The reader then accessed the CAD prompts and reviewed the mammograms to examine areas marked by the CAD system. Any additional findings were recorded, along with a recommendation to recall the patient for further assessment or for the patient to return in 3 years for routine screening. Recommendations for recall were recorded but were not acted upon.

For consistency, the film reading protocols currently used at the two UK National Health Service Breast Screening Programme screening centres (and used in the original 1996 double reading) were retained in this study. In one centre, when double reading, a case was recalled if either reader marked it for recall. With CAD, the single reader decided whether or not to recall. In the other centre, cases were scored on a five-point scale. In double reading, cases scored three or more by either reader were recalled, and those scored one by both readers were not. A case scored two by either reader was discussed by the readers involved or was discussed with another reader and a decision made to recall or not. With CAD, cases scored two were discussed with another reader to determine whether or not they should be recalled. The readers also assessed breast density and marked this on a 10 cm visual analogue scale.

### Ground truth

One researcher (an experienced breast radiologist) from each centre (FJG, CRMB) agreed a protocol for assessing the ground truth. The researchers independently reviewed the mammograms of all of the cancer cases. For each cancer case, prior films and any additional clinical information – including the anatomical location and histopathology of the lesion – were made available and the radiologists reviewed the mammograms according to an agreed protocol. Lesion characteristics and size were recorded *pro forma *and the extent of the boundaries of the cancer was marked on the mammogram as a ROI.

For masses, the boundary of the mass was annotated and the largest diameter (millimetres) of the mass was recorded. For calcifications, a line was drawn around each cluster to include all particles but to exclude normal regions. Classification of particles as a single cluster or as multiple clusters was judged on the basis of the available evidence. The number of calcifications was recorded as one of three categories (<5 calcifications, 5 to 19 calcifications, 20 calcifications or more). For diffuse or widespread abnormalities, the boundary line included all of the abnormal area and asymmetric densities were annotated in a similar fashion.

The location of the CAD marks on the 1996 mammogram was then compared with the location of the histologically proven cancer, and the reader decided whether the prompt was correctly placed within the boundary of the ROI. The readers decided that a prompt was correctly placed only if there were sufficient abnormalities on the prior mammogram to represent at least minimal signs of cancer.

### Measurement of prompt size

The CAD prompt size is related to the algorithm score and is proportional to the degree of probability that a lesion is abnormal [29]. The CAD system supplier provided information to enable prompt sizes (pixels) to be calculated from hard-copy printouts of the algorithm score for each prompt. Prompt sizes were also calculated for the random sample of 10% of the 9,781 normal cases.

### Statistical analysis

The presence, sizes and types of CAD markers were analysed with respect to readers' decisions whether or not to recall cases. Associations between prompt attributes, tumour features, breast density and reader recall decisions were assessed by chi-squared tests. Chi-squared trend tests were used in the case of ordered variables such as prompt size. In analyses of prompt type, those cases for which prompting indicated both mass and calcification (malc) are included as mass prompts. Stata statistical software (version 8.0; Stata Corporation, College Station, TX, USA) was used in the analysis [35].

## Results

### Prompting and recall decision

Prompt data and full outcome data were available for 312 of the 315 cancer cases and for 861 of the random sample of 1,000 normal cases. Table 2 presents prompting tabulated against the recall decision for three categories: normal cases (noncancers), any prompt on mammograms; cancer cases, any prompt on mammograms; and cancer cases, a prompt in the ROI containing a cancer.

**Table 2:** Screening decision by presence of a computer-aided detection (CAD) prompt for normal cases (n = 861) and for cancer cases (n = 315)

CAD prompt	Screening decision		
	
	Recalled	Not recalled	All
Normal cases			
Yes	40 (7)	515 (93)	555 (64)
No	5 (2)	301 (98)	306 (36)
Total	45 (5)	816 (95)	861 (100)
Cancer^a ^cases			
Yes	118 (58)	86 (42)	204 (65)
No	8 (7)	100 (93)	108 (35)
Total	126 (40)	186 (60)	312 (100)
Cancer^a ^cases			
In region of interest	97 (82)	22 (18)	119 (38)
Not in region of interest	29 (15)	164 (85)	193 (62)
Data not recorded	3		3
Total	126 (40)	189 (60)	315 (100)

Data presented as *n *(%). ^a^Cancer cases are reported both for prompts anywhere on the mammogram and for prompts only in the region of interest.

In all three categories presented in Table 2, prompted mammograms were significantly more likely to be marked for recall (*P *< 0.001 in all cases). In the cases for which full screening outcome and prompt data were available, there was at least one prompt in 64% of the normal mammograms. For the 312 cancer cases, 65% cases were prompted – of which 119 (38%) had a prompt in the ROI. The cases with a prompt on the ROI consisted of 80 (94%) of the original screen-detected cancers and 39 (17%) of the subsequent cancers.

In the sample of normal cases, 7% of the prompted mammograms were marked for recall. For cancer cases, 58% of those prompted at any location and 82% of the cases prompted in the ROI were marked for recall by the reader. The reader correctly recalled 29 of the 193 cases (15%) where a CAD prompt was present, but not in the ROI.

In the further analyses below, the focus is on prompts within the ROI (that is, prompts correctly marking cancers).

### Prompt size and recall decision

Table 3 presents the associations between prompt size, prompt type and the final decision to recall. Recall was not significantly associated with the prompt type (mass or calcification) for either cancer cases or normal cases. For normal cases, prompt sizes were significantly associated with recall decision – larger prompts being more likely to result in a decision to recall for both masses and calcifications (*P *= 0.01 for trend in both cases). For cancer cases, larger prompts were significantly associated with a decision to recall for masses (*P *= 0.02 for trend) but not for calcifications alone (*P *= 0.9 for trend).

**Table 3:** Screening decision by prompt type and largest prompt size

Prompt type	Prompt size^a^	Screening decision	
		
		Recall	No recall
Normal^b ^cases			
Calcifications only	Small	2 (3)	70 (97)
	Large	5 (45)	6 (55)
Mass^c^	Small	14 (5)	261 (95)
	Large	19 (10)	178 (90)
Total		40 (7)	515 (93)
Cancer^b ^cases			
Calcifications only	Small	11 (79)	3 (21)
	Large	13 (68)	6 (31)
Mass^c^	Small	17 (71)	7 (29)
	Large	49 (94)	3 (6)
Size or type missing		7	3

Data presented as *n *(%). ^a^Prompt size in pixels. For calcifications: small, <14 pixels; large, ≥ 14 pixels. For masses: small, <18 pixels; large, ≥ 18 pixels. ^b^Normal cases with any prompts anywhere on mammograms and cancer cases with any prompts in the region of interest. ^c^If prompting indicated both mass and calcification, the case was included as a mass.

### Prompting, breast density and detection status

Table 4 presents the prompt results by breast density for normal cases, and by breast density and detection status (original 1996 screen, interval cancer or subsequent screen) for cancer cases. For normal cases there was no significant association of prompts with breast density (*P *= 0.2 for trend). As expected, the average density was significantly greater in the cancer cases (mean ± standard error of the mean, 39 ± 1.2) than in the normal cases (29 ± 0.8).

**Table 4:** Associations with the presence of prompts in the region of interest (ROI)

Study group	Factor	Category	CAD prompt (*n *(%))		Average prompt size	
			
			No	Yes	Mass	Calcification
Normal cases	Breast density	<11%	90 (45)	100 (55)	17	10
		11% to 25%	89 (32)	191 (68)	16	11
		26% to 50%	72 (33)	149 (67)	16	11
		51% to 75%	41 (34)	78 (66)	15	13
		76% or more	14 (34)	27 (66)	16	11
Cancer cases with a prompt in ROI	Breast density^a^	<11%	27 (90)	3 (10)	20	-^b^
		11% to 25%	45 (71)	18 (29)	18	13
		26% to 50%	66 (54)	57 (46)	17	13
		51% to 75%	42 (56)	33 (44)	19	14
		76% or more	13 (68)	6 (32)	19	15
	Detection status^a^	Original 1996 screen	5 (6)	80 (94)	18	14
		Interval cancer (1 year)	4 (57)	3 (43)	-^b^	12
		Interval cancer (2 to 3 years)	29 (69)	13 (31)	15	14

^a^Three cancer cases with a missing prompt location, a further two cases with missing density. ^b^No observations in this category.

For cancer cases, the presence of a prompt in an ROI was significantly associated with breast density (*P *= 0.009 for trend) and with detection status (*P *< 0.001). For cancer cases, prompts were more likely to be present for screen-detected cancers compared with interval cancers and cancers detected at the subsequent screening round. Interestingly, a prompt on the cancer was more likely in mammograms with high breast density. There was no significant difference in the prompt types between dense and nondense breasts.

Prompt size did not appear to be significantly associated with density. In relation to the prompt type there was a significant association of prompt size for calcifications (*P *= 0.01), the size reducing with the temporal distance from the evaluation screen, but there was no significant association of prompt size for masses with detection status.

## Discussion

In the present study we investigated the impact of prompt size and location on reader decision-making in mammography using a large dataset of mammograms that contained a high proportion of normal cases, similar to that found in a screening programme.

For both normal cases and cancer cases, mammograms that had been prompted, especially those with larger prompts, were significantly more likely to be recalled. Readers recalled 58% of cancer cases prompted at any location and 82% of cancer cases with the prompt correctly located in the ROI. This is consistent with the results of Taylor and colleagues, who reported 78% of correctly prompted cancers were recalled [28].

Interestingly, 29 cancer cases in the present study were not prompted by CAD but were marked for recall. Of these cases, eight were screen detected and 21 cases were subsequent cancers. This recall may reflect reader variability, a key issue in screening mammography [4,36,37], or may indicate that participating in a study influenced reader behaviour, making them more vigilant or altering their decision threshold [38-40]. In addition, reader performance could have improved in the 7-year interval since the cases were originally read. The recall also demonstrated that the readers were not misled by the absence of a correctly placed prompt and were using CAD, as intended by the manufacturers, to complement their own interpretational skills.

Several observational studies, albeit in experimental settings, have highlighted the requirement for readers not to become reliant on CAD and to recall a suspicious case even where there is no CAD prompt [16,17,41-43]. There is also clear evidence of a learning curve in the use of CAD during which readers become familiar with the system's performance and gain confidence in readily dismissing false prompts [18,40,44,45].

In normal cases, for both masses and calcifications, there was a trend for cases with larger prompts to be recalled. This would substantiate experimental studies that concluded reader performance could be improved if the readers were able to utilise the CAD algorithm output (determined by the CAD system threshold settings and the characteristics of the lesion), which indicates the probability of malignancy [46,47]. For cancer cases this trend was significant for masses but not for calcifications. CAD algorithms have been shown to have a higher sensitivity and specificity for microcalcifications than for masses [6,8,13,14,16,48-51], and microcalcifications are more likely to be detected by readers on the unprompted film [13]. Furthermore, retrospective evaluation of interval cancers also indicates that masses are more likely to be overlooked then calcifications [9,14,52]. This would imply it is more useful to have a system that encourages correct recall of masses [27].

The present study has also shown that cancers are more likely to be found in patients with dense breasts and that the prompts are more often in the correct ROI, but has also shown in normal cases that prompting is not associated with breast density. This is in contrast to the findings of Destounis and colleagues and of Ho and Lam, who reported a decrease in CAD sensitivity in dense breasts [53,54]. Other studies have reported no difference in CAD sensitivity to microcalcifications but a significantly lower sensitivity for masses in dense breasts [14,55,56]. Our observations suggest that CAD is potentially very useful to improve cancer detection in dense breasts [57]. We are uncertain why there is no similar trend for microcalcification. It may be that the reader recalled microcalcification regardless of the size of the prompt because they believe the case should be biopsied. It is sometimes hard for readers to discriminate between benign and malignant calcification and there is huge overlap in the appearance of microcalcification patterns.

Taylor and colleagues' study showed that when prompts were circled to indicate a higher confidence of malignancy, the reader tended not to overrule the prompt [47]. Further development of CAD software and a more comprehensive understanding of reader behaviour in relation to prompting are required to establish the optimal display of prompt information that will enhance the reader's perception and assessment of an abnormality [44,53,58]. Readers need to retain their sensitivity to act on lesions that are visible but unmarked by CAD and need to be able to confidently dismiss false prompts. This may be partly achieved by displaying the likelihood of malignancy.

A major limitation in the present retrospective, observational study is that it is not possible to separate the effect of the prompt size from the appearance of the cancer on the mammogram [7,13]. More accurate evaluation of the impact of prompt size on reader behaviour would require re-reading the entire dataset with the cancers marked with a single-sized CAD marker. It should, however, be noted that in all CAD studies it is impossible to separate the contribution due to CAD from that of the reader having a second look at the mammogram [38]. There was also no significant correlation between the prompt size and the tumour size (correlation coefficient = 0.10, *P *= 0.4), suggesting that the increased likelihood of recall with a large prompt is not simply due to the latter being correlated with more sinister malignant features. Retrospective classification of the baseline mammograms in relation to minimal signs or incidental findings and correlation with prompts on the ROI would have been more informative since some prompts may have been over incidental findings that had no relevance to the future development of the cancer.

A further limitation of the study is the two different recall systems. The recall systems were not changed as we wished to replicate as closely as possible the previous reading practice in 1996. In both centres, the recall rates at the original screen were similar and single reading with CAD was associated with higher recall rates and higher cancer detection rates in both centres. More importantly, in terms of the results above, in both centres larger prompts on the cancers were associated with higher recall rates – with only slight differences between the centres.

Other limitations are associated with the retrospective design of the study; that is, the readers were aware that the study dataset contained a slightly higher proportion of cancer cases than would be encountered in routine screening, and reader performance and experience may also have improved since the time of the original double reading.

The present is the first published study to examine the impact of prompt size on reader behaviour in a dataset that contained a large number of normal cases and a high proportion of subtle cancers as well as screen-detected cancers. While this study suggests that the variable size prompts may be of value, we recommend a randomised trial is conducted so that this optional display system can be properly evaluated.

## Conclusion

For both normal cases and cancer cases, prompted mammograms were more likely to be recalled, particularly those cases with a larger prompt size. For cancer cases, larger prompts were more likely to be recalled for masses but there was no such association for microcalcifications.

## Abbreviations

CAD: computer-aided detection; ROI: region of interest.

## Competing interests

The authors declare that they have no competing interests.

## Authors' contributions

FJG was the principal investigator, contributing to the study conception, design and conduct, and drafting and editing of the manuscript. SWD contributed to the study concept and design, supervised the statistical analysis and assisted in drafting and editing the manuscript. OFA performed the statistical analysis and assisted in drafting the manuscript. SMA contributed to the study conception and design, and interpretation of results, and assisted in drafting the manuscript. MGCG contributed to the study design and assisted in drafting and editing the manuscript. MAM was responsible for medical informatics, managed data collection in Aberdeen and assisted in drafting the manuscript. PMG was responsible for the conduct of the study and data management in Manchester, and assisted in drafting the manuscript. CRMB was the lead clinician in Manchester, contributing to mammogram reading and assisting in drafting the manuscript. MW, AKJ, NB, UMB, MAG, JJ, RMR, HED, KAD and GI contributed to mammogram reading and assisted in drafting the manuscript. All authors read and approved the final manuscript.
